# The Effects of *Helicobacter pylori* Infection on Gastric Microbiota in Children With Duodenal Ulcer

**DOI:** 10.3389/fmicb.2022.853184

**Published:** 2022-04-25

**Authors:** Wei Zheng, Zhenya Zhu, Jingjing Ying, Gao Long, Bo Chen, Kerong Peng, Fubang Li, Hong Zhao, Mizu Jiang

**Affiliations:** ^1^Department of Gastroenterology, Children’s Hospital, Zhejiang University School of Medicine, National Clinical Research Center for Child Health, National Children’s Regional Medical Center, Hangzhou, China; ^2^Endoscopy Center and Gastrointestinal Laboratory, Children’s Hospital, Zhejiang University School of Medicine, National Clinical Research Center for Child Health, National Children’s Regional Medical Center, Hangzhou, China

**Keywords:** children, duodenal ulcer, gastric microbiota, *Helicobacter pylori*, 16S rRNA

## Abstract

**Background:**

*Helicobacter pylori* (*H. pylori*) infection is the main cause of chronic gastritis and duodenal ulcer in children. Little is known about the effect of *H. pylori* on gastric microbiota in children with duodenal ulcer. This study is aimed at the characteristics of gastric microbiota in children with duodenal ulcer on *H. pylori* infection.

**Methods:**

We studied 23 children diagnosed with duodenal ulcer by gastric endoscopy because of the gastrointestinal symptoms, 15 children were diagnosed with *H. pylori* infection, while 8 children were without *H. pylori* infection. Endoscopic mucosal biopsy samples were obtained for DNA extraction. Microbiomes were analyzed by 16S rRNA profiling and microbial functions were predicted using the software Phylogenetic Investigation of Communities by Reconstruction of Unobserved States (PICRUSt).

**Results:**

Bacterial richness and diversity of gastric microbiota in duodenal ulcer with *H. pylori*-positive were lower than those negative. The gastric microbiota in *H. pylori*-positive group significantly reduced proportions of six phyla and fifteen genera; only *Helicobacter* taxa were more abundant in *H. pylori-*positive group. Co-expression network analysis showed a more complex network of interactions in the *H. pylori-*positive group than that in the *H. pylori-*negative group. For the predicted functions, lower abundance in the pathways of carbohydrate metabolism, signal transduction, amino acid metabolism, and lipid metabolism were found in *H. pylori-*positive group than the *H. pylori-*negative group. *H. pylori* colonization reduces a microbial community with genotoxic potential in the gastric mucosa of children with duodenal ulcer.

**Conclusions:**

The presence of *H. pylori* significantly influences gastric microbiota and results in a lower abundance of multiple taxonomic levels in children with duodenal ulcer. Children with duodenal ulcer exhibit a dysbiotic microbial community with genotoxic potential, which is distinct from that of children with *H. pylori* infection.

**Clinical Trial Registration:**

[http://www.chictr.org.cn], identifier [ChiCTR1800015190].

## Introduction

Before the discovery of *Helicobacter pylori* (*H. pylori*) in 1984 (Marshall and Warren, 1984), the human stomach was considered to be a sterile organ (Beasley et al., 2015). *H. pylori*, a Gram-negative bacterium, is usually acquired in early childhood (He et al., 2016; Mezmale et al., 2020), which can colonize specifically the human stomach and infects about 50% of the population worldwide (Kotilea et al., 2019; Nagata et al., 2021), with reported links to various degrees of gastric mucosal inflammation, such as chronic gastritis, peptic ulcers, and adenocarcinoma (Pellicano et al., 2016; Choi et al., 2020).

The close relationship between *H. pylori* infection and peptic ulcers has long been known. For example, 94% of patients with gastric ulcer and 98% of patients with duodenal ulcer have an associated *H. pylori* infection (Chen et al., 2018), and ulcer recurrence was reduced after *H. pylori* eradication therapy (Sharndama and Mba, 2022). Both innate and adaptive immune responses play a key role in the host’s response to *H. pylori* infection, but the crosstalk between *H. pylori* and the immune system in pediatric patients is far to be fully understood (Mărginean et al., 2021).

In recent years, gastric microecological as a novel perspective has been explored in *H. pylori* infection. Previous studies have stated that *H. pylori* expresses a negative impact on both abundance and diversity of the gastric bacterial community and evidently predominate in the gastric mucosa of infected individuals, while uninfected individuals exhibit a higher degree of biodiversity (Sung et al., 2016; Bravo et al., 2018; Guo et al., 2019; Spiegelhaue et al., 2020). The recent accumulating evidence supports the idea that *H. pylori* infection can significantly reshape the gastric microbiota either through host/microbial interactions or by microbial/microbial interactions (Schulz et al., 2018; Noto et al., 2019). Clinically, *H. pylori-*associated dysbiosis is associated with many digestive diseases (Guo et al., 2020; Rajilic-Stojanovic et al., 2020). Few studies have been done on the persistence of *H. pylori* and related gastric diseases through the influence of gastric microbiota on immune response (Mărginean et al., 2021). Thus, a better understanding of the dialogues between *H. pylori* and other gastric microbial members might offer novel targets for the prevention or treatment of *H. pylori* infection (Tao et al., 2020).

Despite numerous studies addressing the microbiota of the upper gastrointestinal tract (Schulz et al., 2015), these studies are usually performed on adults and a small number of children with *H. pylori* infection. Even though *H. pylori* is the leading cause of duodenal ulcer, there are few studies on the effects of *H. pylori* colonization on the gastric microbiota of children with duodenal ulcer. Therefore, exploring the features of gastric microbiota on *H. pylori* infection in children with duodenal ulcer may provide new clues for treatment. This study aims to investigate the characteristics of gastric microbiota in children with duodenal ulcer according to *H. pylori* status and to evaluate the influence of *H. pylori* colonization on gastric microbiota in children with duodenal ulcer.

## Materials and Methods

### Study Cohort

This study included 23 children (5∼14 years old), who exhibited gastrointestinal symptoms, between January 2018 and August 2018, suggestive of peptic ulcer disease, inclusive of recurrent abdominal discomfort and pain, and dyspepsia. The children, composed of 17 (73.91%) boys and 6 (26.09%) girls, were admitted to the Children’s Hospital of Zhejiang University School of Medicine. The mean age of 23 children was 10.84 ± 3.67 years. Exclusion criteria included a history of acute onset of symptoms, the use of antibiotics, antacids, H_2_ receptor antagonist, proton-pump inhibitor (PPI), bismuth-containing compounds, or nonsteroidal anti-inflammatory drugs (NSAID) in the last 4 weeks. The study protocol was approved by the Medical Ethics Committee in the Children’s Hospital of Zhejiang University School of Medicine (2018-IRB-004). Written informed consent was obtained from legal representatives of the children who participated in the study.

### Gastric Biopsies and *Helicobacter pylori* Testing

Patients underwent esophagogastroduodenoscopy at the Children’s Hospital of Zhejiang University School of Medicine. A total of four pieces of mucosa biopsies used for rapid urease test (RUT), culture, histology, and gastric microbiota studies were performed in the gastric antrum, respectively. Endoscopic findings were also recorded. Gastric mucosa biopsy samples taken from the antrum were preserved in the brain–heart infusion broth (Oxoid, Dardilly, France) with 5% glycerin and sent to the laboratory of Hangzhou Zhiyuan Medical Inspection Institute. The other biopsies were frozen at −80°C until DNA extraction. The homogenate of stomach biopsy specimens was inoculated onto Columbia agar plates (Oxoid) supplemented with 5 % fresh defibrinated sheep blood and kept under microaerophilic conditions (5% O_2_, 10% CO_2_, and 85% N_2_) at 37°C for 3 days. Colonies displaying typical *H. pylori* morphology were selected and identified by Gram staining and urease, oxidase, and catalase activity test. *H. pylori* infection diagnosis was determined using the diagnostic criteria from the study “Consensus on diagnosis and treatment of *H. pylori* infection in children” published in the Chinese Journal of Pediatrics (Subspecialty Group of Gastroenterology, the Society of Pediatrics, Chinese Medical Association, 2015). Based on that study, one of the following four diagnostic criteria can be used to diagnose *H. pylori* infection: 1. positive results from gastric *H. pylori* bacterial culture; 2. positive results from the pathological examination of gastric mucosa biopsy and RUT; 3. in the event of inconsistencies arising between pathological examination of the gastric mucosa and RUT results, non-invasive detection, such as ^13^C urea breath test (UBT) or stool antigen test (SAT) can be performed; 4. positive results from either pathological histology of the gastric mucosa or RUT in the event of peptic ulcer bleeding.

### DNA Extraction

Community microbial genomic DNA was extracted from each biopsy sample using a DNA MiniPrep kit (AXYGEN, Suzhou, China) (Bag et al., 2016). Briefly, the biopsy samples were lysed by incubating the sample in ATL lysis buffer with proteinase K overnight at 56°C and following mechanical lysis with Fastprep instrument (MP Biomedicals, Carlsbad, CA, United States) for 1 min at the level of 6.0 m/s, purified with spin columns, and eluted with 400 μl of buffer AE. The quality and quantity of DNA were measured with Nanodrop (Thermo Fisher Scientific, MA, United States). The extracted DNA was stored at -80°C before use.

### 16S rRNA Sequencing

The V3-V4 hyper-variable regions of the 16S rRNA gene were amplified using a universal primer set (341F: CCTACGGGNGGCWGCAG, 785R: GACTACHVGGGTATCT AATCC) with a barcode. All the template DNAs were normalized to the same concentration. PCR was carried out under conditions described by Caporaso et al. (2011). High-fidelity DNA polymerase: TaKaRa EX Taq was used in PCR. PCR products were separated by electrophoresis in 2% agarose gels, purified with a QIAGEN Gel Extraction Kit (QIAGEN, Germany), and pooled at equal concentrations. Sequencing libraries were generated using a TruSeqR^®^ DNA PCR-Free Sample Preparation Kit (Illumina, United States) following the manufacturer’s recommendations, and index codes were added. Library quality was assessed on the Qubit@ 2.0 Fluorometer (Thermo Scientific) and the Agilent Bioanalyzer 2100 system. The library was sequenced on an IlluminaHiSeq 2500 platform (250-bp paired-end reads) at Novogene Bioinformatics Technology Co., Ltd. (Beijing, China).

### Microbiota-Sequencing Data Analysis

Barcodes as well as forward and reverse primer sequences were removed and raw sequences were analyzed using the Quantitative Insight into Microbial Ecology (QIIME), version 1.9 (Caporaso et al., 2010). Based on the distribution characteristics of low-quality scores of MiSeq sequencing data, quality control of original data was executed. Chimera sequences were removed using the UCHIME algorithm to yield clean tags for further analysis (Edgar et al., 2011). Sequence analysis was performed using UPARSE pipeline version 7.0.1001 (Edgar, 2013), sequences were clustered into operational taxonomic units (OTU) assuming 97% similarity. Representative sequences for each OTU were screened for further annotation. Ribosomal database project (RDP) Classifier (Version 2.2) was used to annotate taxonomic information for each representative sequence based on the Green genes 97% reference data set (McDonald et al., 2012). OTU abundance information was normalized using a standard sequence number corresponding to the sample with the fewest sequences. Subsequent analyses of diversity were performed based on this output-normalized data using QIIME.

### Statistical Analysis

Count data were presented as ratio or proportion, measurement data were presented as means ± SD (X ± SD). Differences were determined by *t*-test, chi-square test, and Kruskal-Wallis test between two groups. Statistically significant differences in the relative abundance of taxa were performed using linear discriminant analysis effect size (LEfSe). A significant alpha at 0.05 and an effect size threshold of 4 were used for all the biomarkers discussed in this study. For the co-occurrence network analysis, OTUs with relative abundance > 0.05% of the microbiome were subjected to Spearman correlation analysis of their occurrence patterns (Barberán et al., 2012; Cardinale et al., 2015), using the non-rarified sequence data. Only correlations [*P* < 0.05 after false discovery rate (FDR) correction] were visualized through network analysis with software Cytoscape 3.6 (Shannon et al., 2003). The functional genes of bacterial communities based on the sequencing data were analyzed by Phylogenetic Investigation of Communities by Reconstruction of Unobserved States (PICRUSt) (Langille et al., 2013). Predicted functional genes were categorized into Clusters of Orthologous Groups (COGs) and Kyoto Encyclopedia of Genes and Genome (KEGG) orthology (KO), and compared across patient groups using Statistical Analysis of Metagenomics Profiles (STAMP) (version 2.1.3) (Parks et al., 2014). All the tests of significance were on two sides, and *P* < 0.05 was considered statistically significant.

## Results

### The Characteristic of Patients

In this study, 23 cases with duodenal ulcer were enrolled in the study group, including 15 cases with *H. pylori* infection, other 8 cases without *H. pylori* infection (Supplementary Table 1). Based on test results and endoscopic findings, patients were divided into two groups: 1. *H. pylori*-positive ulcer group (*n* = 15) [results consistent with *H. pylori* infection diagnosis, endoscopic examination showing duodenal bulbar ulcers, histopathological examination showed various degrees of inflammation]; 2. *H. pylori*-negative ulcer group (*n* = 8) [no *H. pylori* infection, but endoscopic examination showing duodenal bulbar ulcers, histopathological examination showed various degrees of inflammation]. The clinical information of patients including the indication for endoscopy and the main endoscopic finding was presented in Table 1.

**TABLE 1:** Summary of the study subjects’ characteristics.

Clinical information	*H. pylori* positive	*H. pylori* negative	*P*-value
Mean age ± SD (years)	11.23 ± 3.33	10.09 ± 4.38	0.53
Gender *n* (%)			
Male	11 (73.33)	6 (75.0)	0.93
Female	4 (26.67)	2 (25.0)	
Main endoscopic finding *n* (%)			
Esophagitis + duodenal ulcer	2 (13.33)	0 (0)	0.28
Gastritis + duodenal ulcer	13 (86.67)	8 (100)	

### Comparison of Gastric Microbiota

**FIGURE 1:**
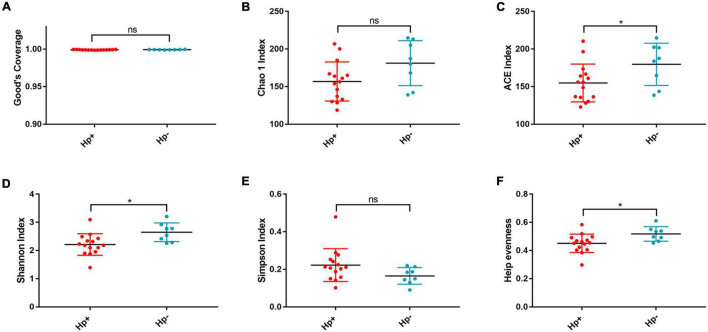
The richness and diversity of the gastric microbiotain children with duodenal ulcer between the *H. pylori*-positive group and the *H. pylori* negative group. Good’s coverage **(A)**, Chao1 **(B)**, ACE **(C)**, Shanon **(D)**, Simpson **(E)**, and Heip evenness **(F)** were used to evaluate the overall structure of the gastric microbiota in the two stomach microtas. ns: *P* > 0.05; *P < 0.05. Hp+, *H. pylori*-positive group; Hp-, *H. pylori*-negative group.

Good’s estimator of coverage was nearly 100%, indicating that the identified reads represented the majority of bacterial sequences present in the stomach (Table 2 and Figure 1A). Based on 16SrRNA bacterial gene sequencing, microbiome profiles in the gastric mucosa of 23 individuals were dominated by phyla Proteobacteria, Campylobacterota, Actinobacteria, Bacteroidetes, Cyanobacteria, and Firmicutes. The bacterial compositions among groups were as follows: Proteobacteria (52.69%), Campylobacterota (26.00%), Actinobacteria (1.55%), Bacteroidetes (0.68%), Cyanobacteria (0.36%), and Firmicutes (0.32%) in *H. pylori-*positive group; Proteobacteria (82.67%), Actinobacteria (4.46%), Cyanobacteria (3.45%), Firmicutes (0.92%), Campylobacterota (0.67%), and Bacteroidetes (0.57%) in *H. pylori-*negative group.

**TABLE 2:** Comparison of phylotype coverage and diversity estimation of the 16S rRNA gene libraries at 97% similarity in *H. pylori* positive group and *H. pylori* negative group.

Coverage and diversity	*H. pylori* positive	*H. pylori* negative	*P*-value
Good’s coverage (%)	99.900 ± 0.033	99.920 ± 0.024	0.156
ACE	154.854 ± 25.133	179.650 ± 28.087	0.042*
Chao1	156.845 ± 25.991	181.180 ± 29.852	0.055
Shannon	2.213 ± 0.380	2.648 ± 0.331	0.013*
Simpson	0.222 ± 0.087	0.165 ± 0.044	0.098
Heip evenness	0.451 ± 0.065	0.518 ± 0.051	0.02*

*P < 0.05.

### Alpha and Beta Diversity

**FIGURE 2:**
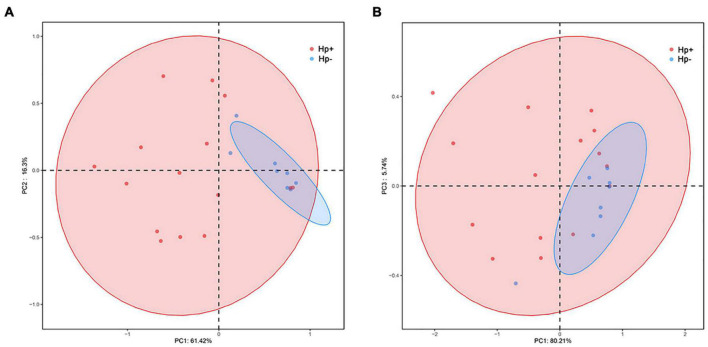
Plots of principal coordinate analysis (PCoA) of the gastric microbiota in the two stomach microbiotas based on the Bray-Curtis distance **(A)** and Weighted Unifrac distance **(B)**. Hp+, *H. pylori*-positive group; Hp-, *H. pylori*-negative group.

Alpha diversity reflects community richness and diversity and they were presented by Chao estimator and ACE estimator and by Shannon index, Simpson index, and Heip evenness, respectively **(**Figures 1B–F). Diversity indices, such as Shannon and Heip evenness, were significantly decreased in *H. pylori-*positive group, while richness indices, such as ACE was also decreased in *H. pylori-*positive group. Beta diversity presented the similarity of community structure and was analyzed using principal coordinates analysis (PCoA) by bacterial abundance clustering. Significant similarity difference was found based on the Bray-Curtis distance between *H. pylori-*positive group and *H. pylori-*negative group (*P* = 0.004). PCoA of weighted Unifrac distances did not show statistical differences (*P* = 0.175). The results of PCoA in the Bray-Curtis and weighted Unifrac distances were shown in Figure 2.

### Altered Gastric Mucosal Microbiota in *Helicobacter pylori* Positive Group

**FIGURE 3:**
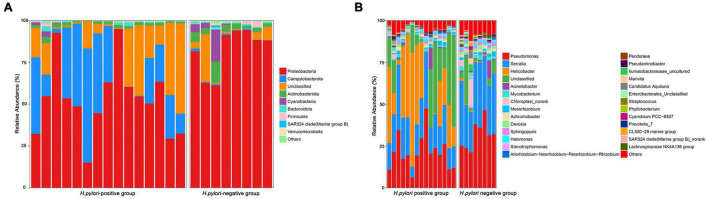
Relative abundance of gastric microbiota at phyla **(A)** level and genera **(B)** level within individual gastric biopsies.

Among the major phyla of gastric microbiota (when mean abundance was greater than 0.1%), clear differences were observed between *H. pylori-*positive group and *H. pylori-*negative group. In total, seven phyla differed in abundance between *H. pylori-*positive group and *H. pylori-*negative group, with Campylobacterota being more abundant in *H. pylori-*positive group (*P* = 0.009), and Actinobacteriota, Gemmatimonadota, Proteobacteria, Verrucomicrobiota, Fusobacteriota and Firmicutes being more abundant in *H. pylori-*negative group (Figure 3A). At the genus level, we expanded the analysis to include the most abundant genera (when mean abundance was greater than 0.1% in *H. pylori-*positive group and/or *H. pylori-*negative group). In total, sixteen genera differed in abundance, with *Helicobacter* being more abundant in *H. pylori-*positive group (*P* = 0.012) and *Pseudomonas*, *Serratia*, *Mycobacterium*, *Sphingopyxis*, *Devosia*, *Pandoraea*, *Hydrogenophaga*, *Caulobacteraceae Unclassified*, *Rhodococcus*, *Gemmatimonas*, *Taonella*, *Phyllobacterium*, *Methylobacterium*, *Methylorubrum* and *Bosea* being more abundant in *H. pylori-*negative group (*P* = 0.003–0.022) (Figure 3B).

**FIGURE 4:**
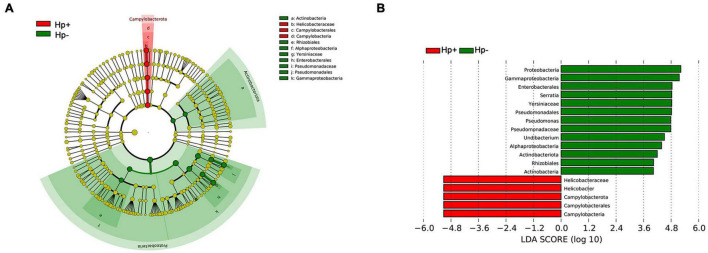
The linear discriminant analysis effect size (LEfSe) identifies the taxa with the greatest differences of gastric microbiota abundance in children with duodenal ulcer between *H. pylori* positive group and *H. pylori* negative group. **(A)** Cladogram of taxonomic distribution in different levels between the two groups. **(B)** The histogram of the linear discriminant analysis (LDA) scores in significantly differential bacteria between the two groups. Hp+, *H. pylori*-positive group; Hp-, *H. pylori*-negative group.

The different bacterial compositions among groups were analyzed by the Metastata algorithm, and biomarkers (the key bacterial members) were further screened by LEfSe analysis. The linear discriminant analysis (LDA) scores showed that Campylobacterota was enriched in *H. pylori-*positive group, Proteobacteria and Actinobacteriota were enriched in *H. pylori-*negative group. At the genus level, *Helicobacter* was the only distinguishing biomarker in *H. pylori-*positive group. In *H. pylori*-negative group, *Serratia*, *Pseudomonas* and *Undibacterium* were significantly increased (Figure 4).

**FIGURE 5:**
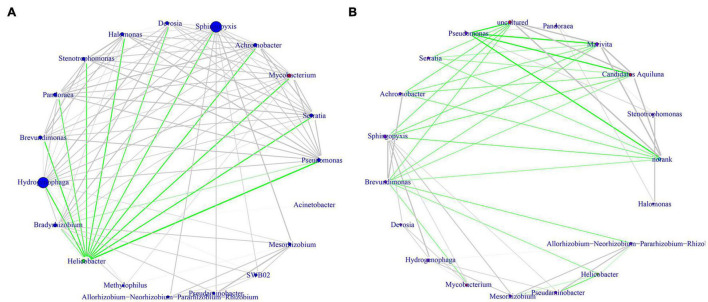
Correlation strengths of the abundant gastric microbiota in different stomach microhabitats from children with duodenal ulcer. Correlation network of the abundant gastric microbiota in *H. pylori*-positive group **(A)** and *H. pylori-*negative group **(B)**. The genera were connected (Gray: positive correlation; Green: negative correlation) when the pair-wise correlation values were significant (*P* < 0.05) after adjusting the *P*-values for multiple comparisons. Furthermore, sub-community detection was performed by placing the genera in the same sub-community (represented by the color of nodes) when many links were found at correlation values > 0.6 between members of the subcommunity.

The overall structure of the gastric microbiota is the result of dynamic interactions between community members. To detect the relationship between different members of the gastric microbial communities, we constructed a network of co-occurrence OTU and interrogated the network for modules using weighted gene co-expression network analysis (WGCNA) (Figure 5). The correlation networks formed different bacterial clusters in the two groups, with a more complex network of interactions in *H. pylori-*positive group than that in *H. pylori-*negative group. In *H. pylori-*positive group, the most dominant member, *Helicobacter* was negatively correlated with *Pseudomonas*, *Serratia*, *Mycobacterium*, *Achromobacter*, *Sphingopyxis*, *Devosia*, *Halomonas*, *Stenotrophomonas*, *Pandoraea*, *Brevundimonas* and *Hydrogenophaga*, those genera demonstrated strong positive correlations. However, most of these correlations were no longer significant in *H. pylori-*negative group, strong negative correlations were formed among *Brevundimonas*, *Sphingopyxis*, *Achromobacter*, *Serratia*, *Pseudomonas*, *Uncultured*, *Marivta*, *Candidatus Aquiluna* and *Norank*.

### Gastric Microbiota With *Helicobacter pylori* Infection Is Characterized by Nitrosating Bacteria

**FIGURE 6:**
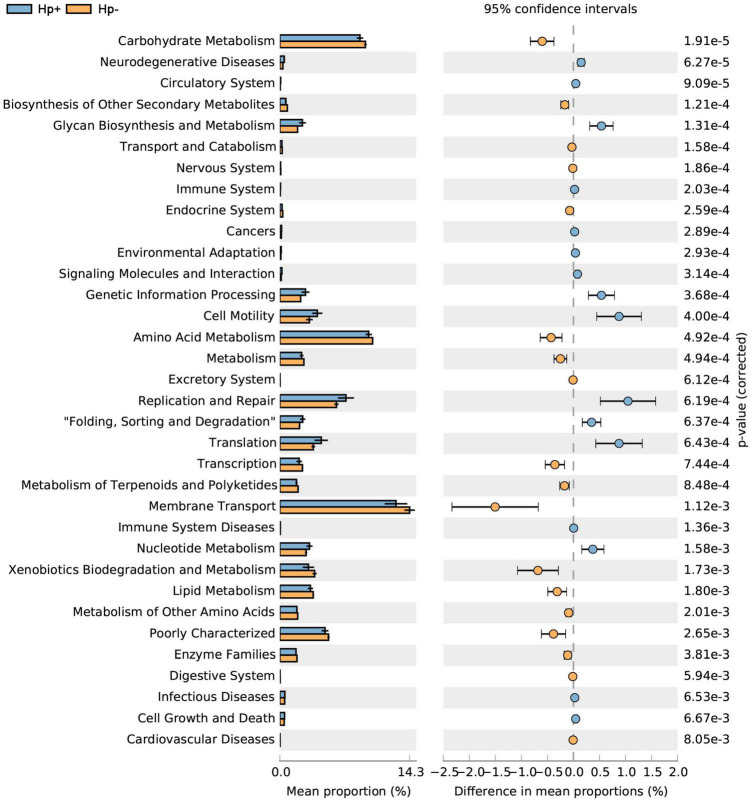
Statistical Analysis of Metagenomics Profiles (STAMP) was used to analyze the different bacterial functions between *H. pylori* positive group and *H. pylori* negative group at the Kyoto Encyclopedia of Genes and Genome (KEGG) level 2. Hp+, *H. pylori* positive group; Hp-, *H. pylori* negative group.

To infer the metagenome functional content based on the microbial community profiles obtained from the 16S rRNA gene sequences we used PICRUSt (Langille et al., 2013). Overall, the gastric microbial communities present in the duodenal ulcer patients with or without *H. pylori* infection could be distinguished based on its functions. The predicted KEGG pathways significantly enriched in *H. pylori*-negative group included carbohydrate metabolism, signal transduction, amino acid metabolism, lipid metabolism, etc. (Table 3, Figure 6, and Supplementary Figure 1).

**TABLE 3:** Predicted Kyoto Encyclopedia of Genes and Genome (KEGG) Pathways differentially abundant between *H. pylori* positive group and *H. pylori* negative group.

KEGG_Pathways	*H. pylori* positive group	*H. pylori* negative group	*P*-values
**meanrel.freq (%)**	**mean rel.freq (%)**	**(corrected)**	
Membrane transport	12.81020	14.31537	0.00111
Carbohydrate metabolism	8.83796	9.43903	0.00002
Transcription	2.13442	2.49131	0.00112
**Xenobiotics biodegradation and metabolism**			
	3.15179	3.83444	0.00173
Poorly characterized	4.98596	5.36991	0.00265
Metabolism	2.39862	2.64914	0.00049
Amino acid metabolism	9.80064	10.23009	0.00049
Lipid metabolism	3.36831	3.68045	0.00180
**Biosynthesis of other secondary metabolites**			
	0.65649	0.82317	0.00012
Enzyme families	1.77929	1.88921	0.00381
Metabolism of other amino acids	1.87500	1.96659	0.00200
Excretory system	0.01603	0.02382	0.00061
Replication and repair	7.29078	6.24300	0.00062
Translation	4.56422	3.68849	0.00064
Genetic information processing	2.82985	2.29201	0.00037
Cell Motility	4.13253	3.25663	0.00040
**Glycan biosynthesis and metabolism**			
	2.48850	1.95081	0.00013
Nucleotide metabolism	3.26567	2.89224	0.00158
Folding, sorting, and degradation	2.52035	2.17161	0.00064
Neurodegenerative diseases	0.48127	0.33344	0.00006
Circulatory system	0.08617	0.04169	0.00009
Cell growth and death	0.51918	0.47573	0.00667
Cancers	0.18649	0.16378	0.00029
**Signaling molecules and interaction**			
	0.22618	0.14939	0.00031
Environmental adaptation	0.15010	0.11174	0.00029
Immune system diseases	0.04799	0.04354	0.00136
Immune system	0.07013	0.04844	0.00020
Endocrine system	0.24280	0.31562	0.00062
**Metabolism of terpenoids and polyketides**			
	1.83871	2.01114	0.00085
Cardiovascular diseases	0.01576	0.02158	0.00805
Infectious diseases	0.55379	0.52832	0.00653
Transport and catabolism	0.22724	0.25596	0.0001
Nervous system	0.08059	0.09231	0.00019
Digestive system	0.02069	0.03328	0.00594

**FIGURE 7:**
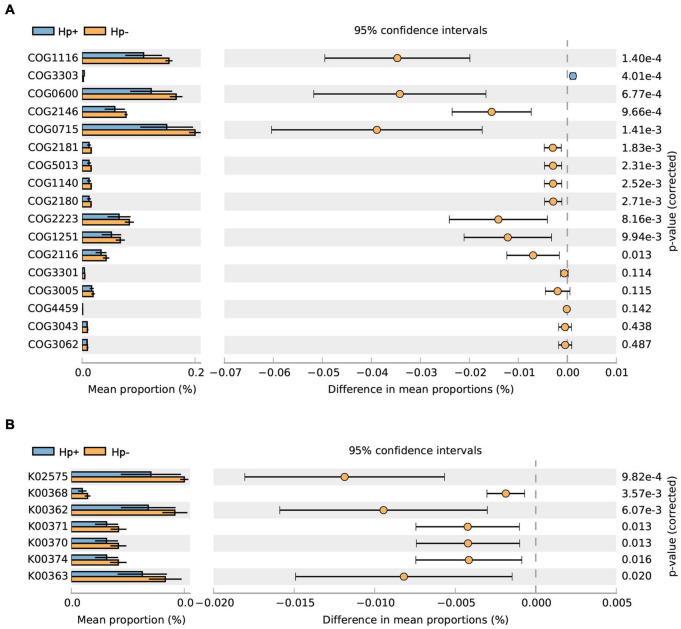
The *H. pylori-*negative group microbiota is characterized by nitrosating bacteria. Functional classification of the predicted metagenome content of the microbiota of *H. pylori-*positive group and *H. pylori-*negative group using **(A)** COG and **(B)** KO. The normalized relative frequency of nitrate reductase and nitrite reductase in patients with *H. pylori-*positive group and *H. pylori-*negative group are shown. Significance was considered for adjusted *P* < 0.05. COG, Clusters of Orthologous Groups; KO, Kyoto Encyclopedia of Genes and Genome orthology; Hp+, *H. pylori* positive group; Hp-, *H. pylori* negative group.

Studies have shown that nitrate-reducing bacterial species contribute to gastric malignant transformation by increasing intragastric concentrations of nitrite and N-nitroso compounds (Ferreira et al., 2018), we next compared *H. pylori*-positive group and *H. pylori*-negative group regarding the microbial functional features involved in those metabolic reactions. The results showed that the functional composition of the total *H. pylori*-negative microbiota had increased nitrate reductase functions, which promote the reduction of nitrate to nitrite, and nitrite reductase functions, which promote the reduction of nitrite to nitric oxide when compared with that of *H. pylori*-positive group (Table 4 and Figure 7). Collectively, these data provide evidence that *H. pylori* colonization reduces a microbial community with genotoxic potential in the gastric mucosa of children with duodenal ulcer.

**TABLE 4:** The selected clusters of orthologous groups (COG) and Kyoto Encyclopedia of Genes and Genome orthology (KO) functions predicted with the whole bacterial community.

Selected COG functions predicted with the whole bacterial community			
#COG	*Z*-Value	*P-*value	Enriched category
COG0600	−3.744	0.0001	*H. pylori* negative group
COG0715	−3.679	0.0001	*H. pylori* negative group
COG1116	−3.744	0.0001	*H. pylori* negative group
COG1140	−3.550	0.0001	*H. pylori* negative group
COG1251	−3.486	0.0001	*H. pylori* negative group
COG2116	−3.422	0.0001	*H. pylori* negative group
COG2146	−3.679	0.0001	*H. pylori* negative group
COG2180	−3.550	0.0001	*H. pylori* negative group
COG2181	−3.551	0.0001	*H. pylori* negative group
COG2223	−3.550	0.0001	*H. pylori* negative group
COG3005	−3.034	0.0001	*H. pylori* negative group
COG3043	−2.776	0.004	*H. pylori* negative group
COG3062	−2.711	0.005	*H. pylori* negative group
COG3301	−2.711	0.005	*H. pylori* negative group
COG3303	−0.291	0.776	
COG4459	−2.969	0.002	*H. pylori* negative group
COG5013	−3.550	0.0001	*H. pylori* negative group

**Selected KO functions predicted with the whole bacterial community**			

**#KO**	***Z*-Value**	***P-*value**	**Enriched category**

K00362	−3.357	0.0001	*H. pylori* negative group
K00363	−3.098	0.0001	*H. pylori* negative group
K00368	−3.679	0.0001	*H. pylori* negative group
K00370	−3.228	0.001	*H. pylori* negative group
K00371	−3.228	0.001	*H. pylori* negative group
K00374	−3.227	0.001	*H. pylori* negative group
K02575	−3.648	0.0001	*H. pylori* negative group
K03385	−0.581	0.591	

*Predicted metagenome of the gastric microbiota using COG in Phylogenetic Investigation of Communities by Reconstruction of Unobserved States (PICRUSt). Predicted COG and KO functions were analysed in STAMP using the two-group comparison with Kruskal-Wallis Test.*

## Discussion

In this study, the gastric microbiota characteristics in children with duodenal ulcer caused by *H. pylori* colonization were investigated through gastric mucosal specimens, which clearly revealed the microecological influence of *H. pylori* infection on the stomach of children with duodenal ulcer. The result showed Proteobacteria, Actinobacteria, Cyanobacteria, Firmicutes, Campylobacterota and Bacteroidetes were the most abundant bacteria in children’s gastric mucosa with and without *H. pylori* infection, in accordance with the previous descriptions (Brawner et al., 2017; Llorca et al., 2017; Miao et al., 2020; Zheng et al., 2021). Campylobacterota became the second dominant bacteria in gastric mucosa with *H. pylori* infection. The richness and diversity of gastric mucosa microbiota were lower in the *H. pylori*-positive children than that of the *H. pylori*-negative children presented by alpha diversity indexes. *Helicobacter*, *Pseudomonas*, *Serratia*, *Mycobacterium*, *Sphingopyxis*, *Devosia*, *Pandoraea*, *Hydrogenophaga*, *Caulobacteraceae Unclassified*, *Rhodococcus*, *Gemmatimonas*, *Taonella*, *Phyllobacterium*, *Methylobacterium*, *Methylorubrum* and *Bosea* were significantly different distinguishing *H. pylori-*positive group from *H. pylori-*negative group at the genus level. Importantly, by applying the LEfSe algorithm that was validated for high-dimensional microbiome data sets, we were able to determine the bacterial taxa that most likely explain differences between the two groups (Ferreira et al., 2018).

*Streptococcus*, *Prevotella* and *Neisseria* were among the most commonly found genera in the adult non-neoplastic stomach (Ferreira et al., 2018), in our study, the contents of the three kinds of microorganisms were very low, and the abundance level was lower than 0.5%. Although a recent study based on children reported significant differences in the abundance levels of *Streptococcus*, *Prevotella* and *Neisseria* between *H. pylori-*positive group and *H. pylori-*negative group (Miao et al., 2020), however, we ended up with a contrasting inference in this study. We believe the underlying reason had to do with various factors such as age, genetics, region and dietary habit.

The LEfSe analysis revealed common genus biomarkers, *Serratia*, *Pseudomonas*, *Undibacterium*, which can be used for differentiating *H. pylori-*positive group from *H. pylori-*negative group. *Serratia* is one of the major bacteria in the stomach of *H. pylori*-negative individuals (Li et al., 2017). *Serratia* is reported to be the most abundant genera in the gastric microbiota of both preterm infants and formula milk-fed infants with a median relative abundance of 15.68% and 34.20%, respectively (Moles et al., 2017; Ku et al., 2020). *Serratia* is often found in the living environment (children’s toys) and pathological conditions (blood infections, skin abscesses, bacterial meningitis, et al.) (Martín-Nalda et al., 2011; Abdinia et al., 2014; Martínez-Bastidas et al., 2014; Johnson et al., 2020). *Pseudomonas* is widely described in the human adult stomach and is regarded as one of the three most common bacterial genera found in the stomach of children with or without *H. pylori* infection (Llorca et al., 2017). In the case of *H. pylori* infection, patients with high *Pseudomonas* abundance tend to have no discomfort such as indigestion (Pereira et al., 2018). *Pseudomonas* favors intestinal translocations with Gram-negative bacteria or their endotoxins and could trigger sepsis, septic shock, secondary peritonitis, or various intestinal infections (Iacob and Iacob, 2019). The neonates with gut dysbiosis showed an increased abundance of *Pseudomonas* and had low levels of short-chain fatty acids in their stools compared to healthy infants (Rao et al., 2020). Few studies have been done on *Undibacterium* and digestive diseases. Studies based on experimental autoimmune encephalomyelitis in rats showed that the more severe the inflammatory reaction, the lower the abundance level of *Undibacterium* (Stanisavljević et al., 2016). The relative abundance of *Undibacterium* decreased in patients with Idiopathic Pulmonary Fibrosis compared with that in the controls (Yoon et al., 2021).

Analysis of microbial interaction network showed *Helicobacter* to be the core node having multiple negative interactions with various genera, by previous descriptions (Hwang et al., 2015; Das et al., 2017; Parsons et al., 2017). The correlation networks formed different bacterial clusters in the two groups, with a more complex network of interactions in *H. pylori-*positive group than that in *H. pylori-*negative group. This is inconsistent with the results of previous studies (Das et al., 2017), which may be due to our study based on duodenal ulcer rather than gastritis, in addition, not all observed positive or negative relationships between organisms may occur in the actual scenario, since these predictions are based on correlations, which may be statistically robust but biologically not feasible (Hwang et al., 2015).

After having analyzed the diversity and composition of the gastric microbiota and microbial interaction network with or without *H. pylori* infection, we addressed the gastric microbial functions based on KO and KEGG database. Results revealed that lower abundance in the pathways of carbohydrate metabolism, signal transduction, amino acid metabolism and lipid metabolism in *H. pylor*i-positive group. Carbohydrate and lipid metabolism pathways were considered to be associated with a significant decrease of Firmicutes and Bacteroidetes, whose genes encode many enzymes that regulate lipid metabolism to maintain energy homeostasis (Hwang et al., 2015; Peng et al., 2017). A previous study reported that higher microbiota diversity was associated with better health (Clemente et al., 2012), the lower microbiota diversity and lower metabolic pathways in *H. pylori*-positive group might be associated with worse health, which may inversely affect children’s growth (Dror and Muhsen, 2016; Taye et al., 2016). We demonstrated that in comparison with *H. pylori-negative* group, *H. pylori-*positive group has reduced nitrate reductase and nitrite reductase functions. This observation is inconsistent with the hypothesis based on adult studies that during carcinogenesis, changes in gastric mucosa led to reduced acid secretion, which enables bacterial growth to reduce nitrate to nitrite, which is a precursor to carcinogenesis of N-nitroso compounds (Correa, 1992; Ferreira et al., 2018). This suggests that the pathological mechanism of *H. pylori* infection in children and adults may be different, and the specific difference needs further study.

It is worth noting that this study had limitations. First, while it has been reported that the gastrointestinal microbiota is dynamic and can be influenced by many external factors such as drugs or diet (He et al., 2016), we excluded subjects with the recent intake of antibiotics, PPI, and NSAID. Also, all the biopsies were taken at a fasting state during endoscopy to minimize the potential influence by meal. Second, as all the cases were selected from the Children’s Hospital of Zhejiang University School of Medicine, there is the possibility of results having confounding variables that are inevitable in all single-centered studies. In view of that, result from large-scale multicenter prospective clinical studies is recommended.

In conclusion, our study found that the gastric microbiota of children with duodenal ulcer was significantly altered with *H. pylori* colonization compared to those without *H. pylori* infection. The function of gastric microbiota changed with the abundance of microbiota. Our results present a comprehensive and novel perspective on microbial communities of the gastric ecosystem, which is of great significance to further study *H. pylori* colonization *in-vivo*.

## Data Availability Statement

The original contributions presented in the study are publicly available. This data can be found here: https://www.ncbi.nlm.nih.gov/bioproject/PRJNA680429.

## Ethics Statement

The studies involving human participants were reviewed and approved by Medical Ethics Committee in the Children’s Hospital of Zhejiang University School Of Medicine. Written informed consent to participate in this study was provided by the participants’ legal guardian/next of kin. Written informed consent was obtained from the individual(s), and minor(s)’ legal guardian/next of kin, for the publication of any potentially identifiable images or data included in this article.

## Author Contributions

WZ and MJ designed the study. WZ, KP, FL, and HZ collected samples. WZ, ZZ, JY, GL, and BC facilitated DNA sequencing. WZ prepared the manuscript. MJ reviewed and edited the final version. All the authors read and approved the final manuscript.

## Conflict of Interest

The authors declare that the research was conducted in the absence of any commercial or financial relationships that could be construed as a potential conflict of interest.

## Publisher’s Note

All claims expressed in this article are solely those of the authors and do not necessarily represent those of their affiliated organizations, or those of the publisher, the editors and the reviewers. Any product that may be evaluated in this article, or claim that may be made by its manufacturer, is not guaranteed or endorsed by the publisher.
