# Supplementary material for: The Effects of Helicobacter pylori Infection on Gastric Microbiota in Children With Duodenal Ulcer
Source: Front Microbiol. 2022 Apr 25;13:853184. doi: 10.3389/fmicb.2022.853184 (PMC9082302; doi:10.3389/fmicb.2022.853184)
Supplement: Supplementary file 1 [file Data_Sheet_1.pdf]

Table S1 Different methods were used to detect the presence of *Helicobacter pylori* (*H. pylori*) in the two groups.

Group	Patient number	*H. pylori* Culture	RUT*	Giemsa staining#	UBT&
H. pylori positive group	15	+	+	+	/
	16	+	+	+	/
	30	+	+	+	/
	37	+	+	+	/
	40	+	+	+	+
	53	-	+	+	+
	61	+	+	+	+
	84	+	+	+	+
	88	+	-	-	/
	106	+	-	-	/
	108	+	-	-	/
	111	+	-	-	/
	112	+	+	-	+
	114	+	+	+	/
	115	+	+	+	/
H.pylori negative group	11	-	-	-	/
	21	-	-	-	/
	26	-	-	-	/
	34	-	-	-	/
	39	-	-	-	/
	89	-	-	-	/
	103	-	-	-	/
	109	-	-	-	/

*: rapid urease test (RUT)

#: The presence of *H. pylori* in tissue sections was identified by hematoxylin - eosin staining and modified Giemsa staining.

&: urea breath test (UBT)

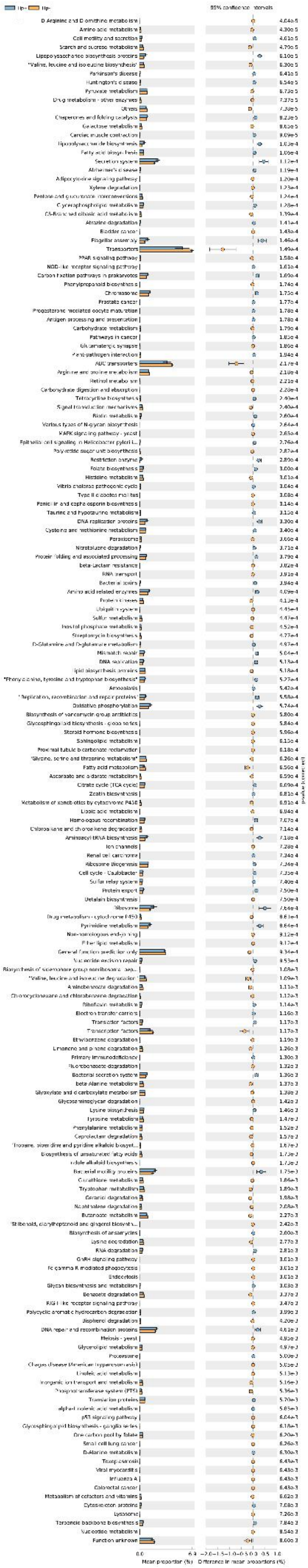

Figure S1. Statistical Analysis of Metagenomics Profiles (STAMP) was used to analyze the different bacterial functions between *H.pylori* positive group and *H.pylori* negative group at the Kyoto Encyclopedia of Genes and Genome (KEGG) level 3.
